# The Route to Autism Spectrum Diagnosis in Pediatric Practice in Bulgaria

**DOI:** 10.3390/diagnostics11010106

**Published:** 2021-01-11

**Authors:** Ivan Ivanov, Iliyana Pacheva, Elena Timova, Ralitsa Iordanova, Fani Galabova, Katerina Gaberova, Aneliya Petkova, Vasil Kotetarov, Margarita Panova, Nikolay Tonchev, Lauren Franz

**Affiliations:** 1 Department of Pediatrics and Medical Genetics, University Hospital “St. George”, 4000 Plovdiv, Bulgaria; ivanovist@gmail.com (I.I.); inapatcheva@gmail.com (I.P.); etimova@gmail.com (E.T.); Ralitsa.Yordanova@mu-plovdiv.bg (R.I.); fani.galabova@gmail.com (F.G.); anelia75@mail.bg (A.P.); margarita.panova@mu-plovdiv.bg (M.P.); 2 Unique Medical Center, 4000 Plovdiv, Bulgaria; tonchev_92@abv.bg; 3 Department of Pediatrics and Medical Genetics, Medical Faculty, Medical University, 4000 Plovdiv, Bulgaria; vasilkotetarov@abv.bg; 4 Department of Psychiatry, University Hospital “St. George”, 4000 Plovdiv, Bulgaria; 5 Duke Center for Autism and Brain Development, Deptment of Psychiatry, Durham, NC 27705, USA; lauren.franz@duke.edu

**Keywords:** autism, neurodevelopment, age of diagnosis, pediatrics

## Abstract

Diagnosis of autism spectrum disorder (ASD) before the age of three years is a challenge. Analyzing the present practice may help reaching that goal. Aim: To investigate developmental abnormalities and diagnostic pathway of ASD patients in pediatric practice. Methods: Retrospective cross-sectional study of 192 children aged 13 months to 17 years 11 months (average 4 years 9 months), investigated in an outpatient and hospital setting from January 2015 to June 2018 by a semi-structured history and clinical examination, and diagnosed with ASD by Diagnostic and Statistical Manual of Mental Disorders, Fifth Edition (DSM-5) criteria. Results: Behavioral peculiarities were detected in the history of the first two years of life in 74.8% of the subjects. The first developmental abnormalities were noticed by the parents at ages from 8 to 36 months (mean 15.6 months) and were predominantly in speech (in 94.6%) and non-verbal communication (11.3%). Developmental regression was reported in 42.1% of the patients occurring between the ages of 6 and 50 months (mean 17.9 months), affecting most commonly speech (88.4% of cases), non-verbal communication (29.2%), and behavior (12.8%). By history, the first manifestations of ASD were noticed at ages from 8 months to 84 months (mean 18.5 months), and were disorders of expressive speech (in 66.7% of cases), receptive speech (in 45.8%), non-verbal communication (35.4%), behavior (27.6%), play (8.9%), socialization (5.7%), and joint attention (2.1%). The most common motive for specialized consultation was delay in language development—in 84.6% of children. The age of ASD diagnosis varied between 12 and 132 months (mean 39.7 months), and the time period between first ASD manifestations and diagnosis was in the range of 0 to 79 months (mean 23.3 months). Many symptoms of abnormal social communication, unnoticed by parents, were detected objectively in more than 95% of the cases—absent or rare spontaneous or reciprocal smile; lack of sharing of interest or affect; abnormal eye contact; lack of finger pointing; lack of gaze to a pointed object; poor facial expressions; lack of imaginary play, etc. Conclusions: Almost two years are needed for diagnosing abnormal development in other domains besides speech in ASD patients. Diagnosis before the age of three years can be achieved by focusing parents’ and pediatricians’ attention on social communication and behavior in patients with speech delay or developmental regression.

## 1. Introduction

Pediatricians and General Practitioners (GPs) are facing an increasing number of infants with impairments, predominantly in the verbal and non-verbal communication and in behavior, suggesting autism spectrum disorder (ASD). The increased attention and interest of pediatricians to ASD is consistent with the worldwide data on its increasing incidence [1], which is attributed to better diagnosis and registration [2], but also admitting absolute increase in the disease burden attributed mainly to environmental factors [3,4].

The latest version of the Diagnostic and Statistical Manual (DSM-5) reflects the significant advances in the understanding of ASD and updates the criteria for this disease [5,6,7]. The use of DSM-5 criteria facilitates early diagnosis because it allows the detection of ASD before the age of three and eliminates the need to differentiate cases in the several entities of pervasive developmental disorders of Classification of diseases ICD-10 [6].

Early diagnosis of ASD provides an opportunity for timely and adequate therapy, and increases the chances of a more successful school education and socialization [8,9]. Factors that may speed up or delay diagnosis are numerous [10] and with great variability country by country, but the basic are maternal education level [11], socio-economic status (doubted as a direct factor) [12], national and regional screening and evaluation policy and tools, qualification of the personnel, funding, social awareness, stigma, etc., [1,13].

A review of the worldwide practice showed that ASD is presently diagnosed by multidisciplinary teams or by skilled individual specialists—pediatricians, developmental pediatricians, child psychiatrists, psychologists, or others—depending on the healthcare system and the resources of each country [1,14].

In Bulgaria, awareness on ASD is in constant rise in the last 10 years, leading to increased referral for diagnostic evaluation, but still there is no universal screening implemented [15]. A study based on interviews of medical professionals in Bulgaria disclosed some potential barriers in the diagnostic process including lack of pediatric psychiatrists [16]. Some instruments, like Modified Checklist for Autism in Toddlers (M-CHAT) and Childhood Autism Rating Scale (CARS) are validated, but still not widely used. Autism Diagnostic Observation Schedule (ADOS) and Social Communication Questionnaire (SCQ) are also used by some psychiatrists [16]. According to that study the mean delay from first ASD symptoms to diagnosis is 14 months [16]. To our knowledge, there is no clinical study that estimated directly the route from first symptoms to diagnosis in pediatric practice, so that to evaluate the gap between intention (the before mentioned questionnaire data) and practice.

## 2. Materials and Methods

The study was retrospective encompassing all children with ASD diagnosed in two connected pediatric settings between January 2015 and June 2018. The study sites provide tertiary medical service in pediatric neurology to patients mainly from southern Bulgaria with population of about 2,500,000. Depending on clinical, social and other variables the patients in this study were treated either in an outpatient service, provided by Unique Medical Center, or as inpatients in the Department of Pediatrics of St. George University Hospital. The staff of these two sites is mostly the same, and the principles and practice of clinical work are the same. Thus, combining the two sites in one study decreases selection bias.

Inclusion criteria were ASD according to the criteria of DSM-5. Exclusion criterion was incomplete documentation.

Of the 204 children diagnosed with ASD, 12 were excluded due to incomplete data. The remaining 192 were the subjects of the study. The average age at first examination by the study team was 4 years 9 months (from 13 months to 17 years 11 months). Characteristics of the study participants is provided in Table 1. No reliable data on socio-economic status and parental education are available.

The 39 syndromic cases included cases with 14 proven etiology: 6 with dysmorphism with or without additional features and chromosomal aberrations (3q29 microdeletion; 14q32.2 duplication; inv(9)(p12q13); t(15q11; 2q13.3); 47XУУ a complex case with multiple chromosomal rearrangements); 3 monogenic syndromes (2 with tuberous sclerosis; 1 with neurofibromatosis type1); 4 cases with brain structural lesions (1 with perisylvian polymicrogyria; 3 with ante- or perinatal lesions with atrophy, gliosis, or cysts); 1 case with epileptic encephalopathy (Lennox–Gastaut syndrome). Another 23 cases were classified as probably syndromic: 10 cases were grouped as with probable chromosome aberration because of marked dysmorphism and positive familial history or intrauterine growth retardation; in 7 cases monogenic syndromes with suggested clinically (2 cases with dysmorphism suggestive of fragile X syndrome; 2 cases compatible with PTEN mutation; 2 cases with tuberous sclerosis skin signs; 1 case with Rett syndrome phenotype); 5 cases had unspecific brain lesions or without proven cause (2 with brain atrophy; 1 with periventricular leukomalacia; 1 with isolated pituitary gland hypoplasia; 1 with external hydrocephalus); 2 cases with probable mitochondrial disorder (hyperlactacidemia with elevated alanin); 1 case with suspected epileptic encephalopathy due to electric status epilepticus in slow wave sleep). All other 153 cases were classified as idiopathic.

The methodology of the study included:

(1) Structured questionnaire for the history of pregnancy, birth and neurodevelopment;
(2) Structured questionnaire on behavioral peculiarities
(3) Semi-structured history on parental neurodevelopmental concerns that motivated their visit.
(4) Semi-structured history and examination for ASD according to previously describes methodology [17] that included observation and parental information on: spontaneous behavior; spontaneous play; attitude to toy-cars, building blocks, puzzles, books, pencil and paper; attitude to parents; attitude to the physician; joint attention tests—looking at a pointed picture, pointing towards wanted or interesting object; nonverbal communication tests—understanding of gestures for ”Take that!”, “Give me!”, “Come here!”, etc.; spontaneous speech—idiosyncratic, melody, prosody, content, grammar, vocabulary, echolalia, etc.; verbal communication—understands commands, verbal production.
(5) ASD diagnostic checklist based on DSM-5 criteria and filled in with the data from the objective examination and the history (list of signs is given in Table 2; additional signs observed or in history were also recorded)
(6) Childhood Autism Rating Scale (CARS)
(7) Modified Checklist for Autism in Toddlers (M-CHAT) [18].

Diagnosis of ASD was reached in three stages: 1. initial assessment, performed by a pediatric neurologist (R.I., F.G., K.G.), fulfilling items from 1 to 7 of the methodology; 2. reassessment of the same items by an experienced pediatrician—pediatric neurologist (I.I.,I.P.,M.P.); 3. confirmation by a multidisciplinary team, including a child psychiatrist (V.K.) and psychologist (E.T., A.P.). The duration of each examination in stage 1 and 2 lasted between 60 and 90 (rarely 120) minutes.

All patients had clinical, neurological and neurodevelopmental examination. Depending on clinical presentation, setting and parental decision, brain imaging (MRI or CT) was done on 41 cases, metabolic screening on 29, and genetic studies on 46 patients.

### Statistical Analysis

The following methods of statistical analysis were used: descriptive statistics; percentage distribution; Chi-square, *t*-test and correlation analysis. All tests were applied assuming 5% confidence level for the null hypothesis (*p* = 0.05). All calculations were performed using the statistical software EpiCalc 2000 (http://www.brixtonhealth.com/epicalc.html, accessed on 10 February 2019) and Microsoft Corporation. Microsoft Excel [Internet]. 2018. Available from: https://office.microsoft.com/excelThe study was approved by the Scientific Ethics Committee of Medical University-Plovdiv – ID5 (17 June 2020).

## 3. Results

### 3.1. Passport and Premorbid Anamnestic Data

Most of the 192 participants had uneventful history: normal pregnancy in 113 (58.8%; missing data for 2 children); maternal age at birth less than 36 years—155 (89.1%; 18 without data); born at term—155 (89.9%; 18 without data); normal birth weight—154 (96.2%, 32 without data); normal birth and early neonatal period—148 (80.1%, without data for 9 children). The family history was positive for developmental delay (mainly in language) or intellectual deficit in 76 patients (39.7%; 1 without data).

### 3.2. Behavior Peculiarities

The use of a structured questionnaire on behavioral peculiarities of 131 patients in their first two years of life showed the following frequencies according to the retrospective parental data, taken during the first interview of our team: prefers to play alone—62 children (47.3%); rarely smiles—37 (28.2%); over-crying (difficult to calm down)—31 (23.7%); too quiet—24 (18.3%); did not respond to communication attempts—22 (16.8%); did not respond when called by name—4 (3.0%). A total of six children were reported positive for any 6 of these 7 signs; any 5 signs were observed in 85 children; 4—in 23 children; 3—in 17; 2—in 2, only 1 sign—in 22 children. None of these behavioral features were reported in 33 children (25.2%).

### 3.3. First Impairments in Neurodevelopment

The parents reported retrospectively at the time of examination that first sings of neurodevelopmental impairment were noticed on average at 15.6 months (8 to 36 months; no data in 8 children). These first signs were in the domains of expressive and receptive language in 175 children (94.6%), non-verbal communication in 21 children (11.3%), behavior in 18 (9.7%), motor development in 15 cases (8.1%) and food selectivity in 1 child (0.5%). Data on the type of the first neurodevelopmental impairment were missing for 7 children. In most children (148; 80.0%), the first impairments were only in one domain, most often language development. In 29 children, two domains were affected (15.7%) and 8 in three domains (4.3%).

### 3.4. Neurodevelopmental Regression

Regression in neurodevelopment was reported by the parents of 79 children (42.1%; 6 children with missing data). The regression occurred on average at 17.9 months of age (from 6 to 50 months).

The areas or presentations of regression were in: speech development—69 patients (88.4% of the 78 cases with detailed data on regression); non-verbal communication—23 patients (29.2%); behavior—10 patients (12.8%); motor development—3 patients (3.8%); food selectivity—2 patients (2.6%); affective seizures—1 patient (1.3%). The regression most often affected one neurodevelopmental domain (51 children, 65.4%), and less frequently in two or more domains—27 children (34.6%).

Regression was preceded by intercurrent infection (8 of 78 cases, 10.2%), vaccination (also 8 children, 10.2%), trauma (3 patients, 3.8%), surgery (2 children, 2.6%) or febrile epileptic status (1child, 1.3%). For the majority of these children (56; 71.8%) no provoking factor was identified. The clinical presentation of patients with regression after vaccination was not statistically different form that of other patients with or without regression (Supplementary 1).

Post-regression improvement was described by the parents of 16 (23.9%) of the 67 children with available data on neurodevelopment after regression. For 7 of these children, improvement started on average at age 28.8 months (between 18 to 48 months). The majority of children (51; 76.2%) were without any data of improvement after regression.

### 3.5. First ASD Symptoms

The analysis of the parental data on the first ASD symptoms revealed multiple manifestations. The first three reported for each child were summarized and presented in Table 2. Delay in expressive and receptive language was included in the list, albeit not being in the DSM-5 criteria.

The age of appearance of these first ASD symptom in 174 of the children was 18.5 months (from 8 to 84 months). In 10 children there were no data on the age of onset of the first ASD symptoms.

### 3.6. Reasons for Consulting a Specialist

The reasons for seeking medical help were: delay in language development—154 children (84.6.2% of 182 patients with relevant data); language regression—34 (18.7%); delay in communication development—24 (13.2%); communication regression—11 (6.0%); behavioral abnormalities—56 children (30.8%); impairment in the interaction with children—8 (4.4%); other manifestations—8 (4.4%). In 91 (50.0%) children, two or more of the above abnormalities were the cause to seek medical attention, and in the remaining 91 children (50.0%) the first ASD symptom that lead to ASD diagnosis was only one, and it was most frequently language delay—79 children (43.4%).

### 3.7. ASD Diagnosis by Age, Specialist, and Delay

The average age of ASD diagnosis was 39.7 months (from 12 to 132 months), based on the available data of 178 patients. ASD had been diagnosed firstly by a pediatric neurologist in 135 children, by a psychiatrist or a child psychiatrist—in 33, or by an unknown number and type of specialists—in 10 children.

The youngest age of ASD diagnosis was at 12 months and it referred to a patient with macrocephaly, dysmorphism, normal neurological status, severe emotional-social and language delay—not responding on calling by name, not fulfilling commands, not seeking help or attention, no social smile, interested only in his own toy which he chewed, soothed only after switching on the fountain tap; speech consisted of mooing monotonous sounds; frequent stereotypes with hand waving. These symptoms persisted in the last follow-up visit at 4 years 10 months.

**Figure 1:**
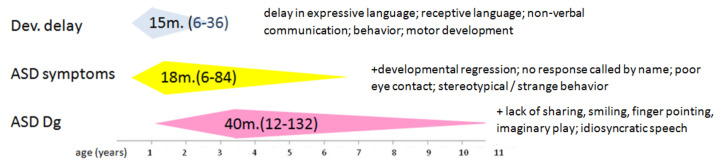
Age of appearance of the first signs of developmental delay, of ASD and of diagnosis of ASD (mean age in months (min-max). The first most common signs and symptoms of developmental delay and ASD observed by parents, and also by physicians at ASD diagnosis are added on the right of each step. Each next step includes also the preceding symptoms.

The delay from the age of occurrence of the first neurodevelopmental impairment to the age of ASD diagnosis was mean 25.1 months (from 0 to 114 months) (Figure 1).

The mean delay from the onset of the first ASD symptoms to ASD diagnosis was 23.3 months (0 to 79 months).

The delay in ASD diagnosis is greater in patients diagnosed at older age (*R* = 0.89) and in patients from rural area (mean delay 28.2 months; range—0–72 months) compared to urban living (21.5 months; 0–79 months; *p* < 0.05). Preterm children with ASD were diagnosed at mean age 15.9 months (range 0-42 months), while the rest were diagnosed at 24.0 months (0–79 months; *p* = 0.05). Delay in ASD diagnosis was not found to correlate with sex, etiology (idiopathic vs. syndromic), age of perceiving first symptoms of neurodevelopmental delay or of autism, evidence of regression, or family history of neuropsychiatric abnormality. A weak negative correlation was found between delay in ASD diagnosis and Intelligence Quotient (IQ) or Developmental Quotient (DQ) (−0.42), the IQ and DQ being lower in syndromc cases (40.9, range 18–72) than in idiopathic cases (mean 56.8, range 9 to 107).

### 3.8. ASD without Language Delay at Diagnosis

These 3 cases were 2 boys and 1 girl and during their last examination at 3, 11, or 13 years no language delay was found. History revealed town residence, no data of prematurity, imtrauterine growth retardation, abnormal birth, or familial history of abnormal development. Behavioral peculiarities had been noticed in two of them and were few (1 or 3 of them). The first impairments in neurodevelopment were language delay in 2 patients and motor coordination delay in one cases, noticed between 9 and 18 months of age (mean 17.7 months). The first symptoms of ASD were impairments in social communication, noticed between 12 and 84 months (mean 44.0 months), later than the rest of study cases (mean 18.1 m., range 9–72 m., *p* < 0.05). Diagnosis of ASD was also later (mean 82.7 m., range 32–132 m.) than the others (mean 39.1 m., range 12–103, *p* < 0.05). Delay of ASD diagnosis was also greater—65.0 m. (23–124 m.) compared to 24.2 m. (6–76 m., *p* < 0.05). Neither patient had intellectual deficit. One suffered from rolandic epilepsy, but without electric status epilepticus in sleep.

### 3.9. ASD Symptoms

In 94 children examined after 1 August 2016, a structured ASD questionnaire based on DSM-5 was filled in with information of the objective examination and history [16] (Table 3). The average age of filling this form was 44.3 months (18 to 120 months).

### 3.10. CARS

CARS was administered to 109 outpatient children. The average grade is 37.5 points (27 to 53). In 4 patients, the total score was below 30—27, 27, 28, and 29. There was no correlation between the CARS score and the delay in diagnosis.

### 3.11. M-CHAT

M-CHAT was administered to 20 children from 1 July 2015 to 1 March 2016. The average number of positive items is 9.8 (from 3 to 20) and for the critical ones—3.0 (0–6). All children in the study showed abnormal M-CHAT results.

## 4. Discussion

### 4.1. ASD Diagnosis

The contemporary American Academy of Pediatrics (AAP) guidelines suggest monitoring, screening and diagnostic evaluation with validated tools as steps that provide timely and accurate ASD diagnosis [13]. Still in many countries the screening and evaluation tests are not translated and validated, and the organizational network that should perform proper monitoring is not efficient enough [15]. In these instances, the clinical skills of the neurodevelopmental specialists are of utmost importance. Direct application of the DSM-5 criteria in clinical practice by physicians experienced in the pathology of child development is possible [19]. Careful structured and patient objective status, coupled with an analysis and explanation of behavior, and backed by history, provides a sound base for ASD diagnosis [1]. Diagnosis based on a structured questionnaire and observation similar to ours has also been applied by other authors [14,20]. In many countries, diagnosis of ASD is made only by experienced pediatricians, pediatric neurologists, clinical psychologists, and other specialists [1], which saves time and resources and allows early initiation of therapeutic intervention, even when diagnosis is uncertain [9]. In addition, the examination by a pediatric neurologist or pediatrician enables the identification of cases of syndromic autism that account for more than 10% of all children with ASD [21]. Consultation with a child psychiatrist and evaluation by a multidisciplinary team are mandatory for difficult to diagnose cases [5]. Thus, the methodology of the present study, with direct application of the DSM-5 criteria by an experienced team, makes the best use of the available resources.

M-CHAT is a recommended and widely used screening tool for ASD [13]. All patients were screened positive in this study, supporting the high positive predictive value of M-CHAT.

CARS is a commonly used diagnostic tool based on previous versions of DSM and includes a summary assessment of dysfunctions from objective and history data with sensitivity 0.89–0.94 and specificity 0.61–1 [1]. The 4 cases in the present study with fewer points than the CARS diagnostic threshold of 30 points support the proposal the cut-off point to be reduced to 25 [22]. This may be even more actual with the DSM-5 criteria, as the construct validity and internal consistency of the CARS in the context of DSM-5 criteria is already approved by some authors [23].

### 4.2. ASD Group Characteristics

In clinical terms, ASD is 4–5 times more frequent in males [24,25], with the difference being greater in older patients [26,27]. These data are consistent with the prevalence of boys in our study. Absence of abnormalities in pre- and perinatal history in most children corresponds to the idiopathic etiology in about 90% of ASD [21].

A large number of ASD children have behavioral peculiarities (developmentally inadequate behavior) in the first years of life in the areas of social interaction, visual and joined attention, stereotypic behavior, language development, etc., [28,29,30,31,32]. In this study, parents of three-quarters of the children reported retrospectively for such peculiarities in behavior in the first year. The most commonly reported behaviors might be related to deficiencies in social interaction (prefers to play alone, smiles rarely, difficult to soothe, not reacting to communication attempts, not responding when called), but others could be interpreted as insistence on sameness, i.e., reduced number of changes in posture (“a too quiet child”) [29].

Impairment in language development is a hallmark of ASD [8]. Although not included as a criterion in the DSM-5, it is a specifier to ASD diagnosis [6]. In 94% of the present cases delay in expressive or receptive language was the first and most often the only symptom that was assumed by parents as concerning in neurodevelopment. This overwhelming proportion of language impairment in our patients implies that a number cases without overt language delay are not diagnosed timely or at all. This suggestion is supported by the greater delay in ASD diagnosis of our three cases without language impairment at time of diagnosis. Detailed history of language delay, although not evident at the moment, may ease timely ASD diagnosis.

Neurodevelopmental regression, mainly in verbal and non-verbal communication, is observed in 10 to 50% of ASD patients [19], most commonly between the ages of 1 and 3 years [8,19]. In this study, we confirmed the high rate of regression (42%) and revealed the most frequently affected domain, language, and the adverse prognostic significance of regression, evolution towards improvement, in only 29% of children with regression. The lack of a provocative factor in more than half of the cases and the similar proportions and disease course in cases with preceding infection or immunization are important data for the general public, suggesting that the effect of vaccine is no different from other intercurrent infections, or is probably nihil as patients without putatively provocative factors or without regression have similar disease course and characteristics, as it is found in this study.

The dissociation between parental opinion about child’s problems and objective status is common in ASD [33]. Parental collaboration in the diagnostic evaluation is rated by Bulgarian physicians as moderate—3, 2–3, 4 in a 1 to 5 scale [16]. Delay in language development was the most common (or even the only symptom in half of the cases) that raised parental concerns and urged them to seek medical help. A number of major symptoms of ASD, such as abnormal visual contact or impaired joined attention, were reported in the history of only 35 and 2% of our cases, respectively, while they were observed during the clinical examination in over 96% of the cases.

### 4.3. Age of Diagnosis

ASD can be reliably diagnosed after 24 months of age and should not be diagnosed beyond 36 months of age [34,35]. The most recent AAP recommendation states that ASD can be diagnosed as young as 18 months of age [13]. Greater public awareness and medical attention on communication and behavior during visits are major factors in order to meet the recommendation to begin therapeutic intervention at 15 months of age [36]. A literature review showed that 75 to 95% of patients with ASD diagnosed earlier that the age of 36 months retain that diagnosis later [29]. The DSM-5 does not set an age limit for diagnosing ASD in the presence of persuasive symptoms, which allows diagnosis at a much earlier age. Moreover, even if the striving for timely and specific therapeutic intervention may lead to overdiagnosis of ASD, this risk may be accepted against the option of exact but late diagnosis.

The actual age of diagnosing autism worldwide ranges from 36 to 114 months (average 48 months) according to data from 36 studies after 2000 [34]. There is a stable tendency of reduction in that age [12], i.e., the average age of diagnosis in the United States of children born in 2007–2010 is already 2.7 or 2.9 years, depending on the presence or absence of intellectual deficits [12].

The average age of ASD diagnosis in this present study was 39.7 months, which is lower than the world average [34], but not fulfilling the requirement of diagnosis before 36 months of age. The actual delay occurred to be much longer than the 23 months perceived by the physicians according the questionnaire survey in Bulgaria [16]. Other authors also think that although the majority of parents of children with ASD report concerns about their child’s development during the first 2 years of the child’s life, the median age of diagnosis is actually after the child’s fourth birthday [37]. How to explain this delay? Several findings of the present study are worth mentioning:

- The 7-months delay of patients living in villages may replace, to some extent, the missing data on education and socio-economic status, assuming that there is some inferiority of these variables in rural inhabitants compared to urban ones.
- The smaller delay in preterm babies, which are regularly and actively followed-up, combined with the absence of correlation between delay magnitude and severity of autism, reflected in CARS, proves the yield of proactive monitoring of neurodevelopmental issues.
- Most parents perceive initially that their children have only language problems, which are thought as “benign, self-healing”. They overlook nonverbal socio-communicative and behavioral problems. These assumptions and the imminent stigma lead frequently to a delay in the search of medical help and cause search of second, third, etc., opinion instead of starting intervention.

### 4.4. Limitations of the Study

The retrospective design of the study limits the validity of the exact ages of symptom onset. Despite that, there is no doubt in the discrepancy between the parental concerns that were mainly in the language domain, and the overwhelming problems in non-verbal communication and restricted-repetitive behavior observed objectively.

ASD diagnosis was made without the support of validated tools. The comprehensive examinations and the approval by child psychiatrist and psychologist made ASD diagnosis firm. We admit that some mild cases might be missed and not included in the study, especially without language delay.

Some answers in the questionnaire of behavioral peculiarities may be not correct due to oblivion and change in the parental assessment of behavior. Despite that, paying attention on this developmentally inadequate behavior may ease early diagnosis.

The high variability in the etiology of syndromic cases and the relatively small number of cases in each etiological entity does not allow us to produce statistically significant conclusions on the disease course of each specific cause. The degree of developmental delay or intellectual impairment may be an universal predictor of the diagnostic delay, with the more severe cases diagnosed earlier, independent of the cause.

## 5. Conclusions

In conclusion, the reduction of the two-year interval between the onset of the first symptoms of ASD and its diagnosis can be achieved by focusing attention to children with atypical social communication, language and behavior in the first two years, or with regression in development. Greater awareness of the general public with stigma reduction, raised sensitivity of pediatricians and general practitioners towards ASD symptoms, and regular monitoring, screening and evaluation with validated tools are the major prerequisites for early ASD diagnosis—before the age of three.

## Figures and Tables

**Table 1:** Characteristics of the study participants.

Variable		*n*	%
Sex	Male	149	77.6
	Female	43	22.4
Habitation	Urban	170	88.5
	Rural	22	11.5
Etiology	Idiopathic	153	79.7
	Syndromic	39	20.3
Developmental Delay/Intelectial Dysability (DD/ID)	Absent	34	21.9
	Mild	58	37.4
	Moderate	40	25.8
	Severe	16	10.3
	Profound	7	4.5
	unmeasurable	37	-
Language impairment	Present	189	98.4
	Absent	3	1.6
Epilepsy	Present	33	17.2
	Absent	159	82.8
Cerebral palsy	Present	5	2.6
	Absent	187	97.4

**Table 2:** First signs of ASD by parental history (*n* = 192).

ASD signs grouped in domains	*n*	%
**Impairment of expressive language**	**128**	**66.7**
language delay	82	42.7
language regression, including in babbling	35	18.2
does not engage in dialogue	1	0.5
idiosyncratic speech	4	2.1
Echolalia	2	1.0
monotonous vocalization or speech	3	1.6
speech stop	1	0.5
**Receptive language impairment**	**88**	**45.8**
does not turn when called by name, or turns rarely	78	40.6
does not execute simple commands	6	3.1
does not pay attention to conversation	2	1.0
late or missing visual search for an asked item	2	1.0
**Impairment of non-verbal communication**	**72**	**37.5**
eye contact—inconsistent, weak or missing	68	35.4
no reciprocal smile	1	0.5
stopped smiling	1	0.5
no facial expression	1	0.5
started waving goodbye late- after 2 years	1	0.5
**Behavioral impairment**	**53**	**27.6**
stereotypical behavior, spins or touched toys, rotates, etc.	29	15.1
episodes of strange behavior	10	5.2
aggressive behavior	5	2.6
affective spells with/without provocation	5	2.6
Hyperactivity	2	1.0
“talk to itself in front of the mirror”	1	0.5
compulsive behavior	1	0.5
**Abnormal play**	**17**	**8.9**
plays alone	5	2.6
does not play with children	3	1.6
monotonous play	2	1.0
licks toys or food	2	1.0
plays mostly with older ones	1	0.5
looks at the angles of objects	1	0.5
prefers playing with small balls	1	0.5
arranges toys in a circle	1	0.5
arranges toys	1	0.5
**Restrictive interests**	**15**	**7.8**
selectivity for food	11	5.7
no interest in new toys	2	1.0
does not want to be photographed	1	0.5
does not want to be touched	1	0.5
**Abnormal or delayed communication development**	**11**	**5.7**
“disturbed communication”	5	2.6
“gets upset when there are many people around”	1	0.5
not interested in children	1	0.5
hugs strangers	1	0.5
“became cool with no emotions”	1	0.5
difficult communication with peers	1	0.5
“impaired social integration”	1	0.5
**Impaired joint attention**	**4**	**2.1**
does not point with a finger	2	1.0
does not look in a pointed direction, or looks briefly	2	1.0

**Table 3:** DSM-5 symptoms of ASD and relevant clinical signs observed during the clinical examination or reported by parents (*n* = 94).

Criteria (A and B), symptoms (1–4) and signs:	*n*	%
A. Persistent deficits in social communication and social interaction across multiple contexts currently or by history:	94	100.0
**1 Deficit in socio-emotional reciprocity:**	**94**	**100.0**
Missing or infrequent spontaneous or reciprocal smile	89	94.7
Lack of sharing emotion, interest or affect	92	97.9
Lack of interaction like give, show, request	93	98.9
Emotionally cool to loved ones	87	92.6
**2. Deficits in nonverbal communicative behaviors used for social interaction:**	**94**	**100.0**
Abnormal visual contact	92	97.9
Failure to look in the finger-pointed direction	92	97.9
Lack of pointing with a finger to a wanted or interesting item	91	96.8
Poor facial expressions	87	92.6
Deficit in understanding gestures	90	95.7
Deficit in the use of gestures	90	95.7
Violation of privacy	82	87.2
3. Deficits in developing, maintaining and understanding relationships:	94	100.0
Missing friendships	84	89.4
Lack of interest in peers	89	94.7
Prefers playing with older or younger	89	94.7
Lonely game	88	93.6
Lack of imaginary play (imitation of telephone conversation, driving a car, feeding a doll, etc.)	90	95.7
Lack of social reference (e.g., orientation by mother’s facial expression)	89	94.7
B. Restricted, repetitive patterns of behavior, interests, or activities currently or by history:	*n*	%
**1. Stereotyped or repetitive movements, use of objects, or speech:**	**92**	**97.9**
Simple motor stereotypes	90	95.7
Arranges toys	87	92.6
Rotates objects	86	91.5
Monotone play	90	95.7
Lack of functional play	89	94.7
Idiosyncratic speech	90	95.7
Echolalia	89	94.7
Perseverations	80	85.1
**2. Insistence on sameness, inflexible adherence to routines, or ritualized patterns of verbal or nonverbal behavior**	**89**	**94.7**
Prefers the same route when driven by car or walking	85	90.4
Prefers the same food	87	92.6
Extreme distress after small changes	63	67.0
**3. Highly restricted, fixated interests that are abnormal in intensity or focus:**	**93**	**98.9**
Strong attachment to unusual objects	83	88.3
Lack of interest in reading books or telling stories	89	94.7
Lack or brief interest in new toys	87	92.6
**4. Hyper- or hyporeactivity to sensory input or unusual interest in sensory aspects of the environment:**	**89**	**94.7**
No response to a call by name	59	62.8
Closure of the ears with the hands in the absence of loud sound	64	68.1
No reaction to pain or fever	57	60.6
Excessive smelling or touching objects	57	60.6
Fascinated by light or movement	56	59.6
Other manifestations	4	4.3

## Data Availability

Data sharing not applicable.

## Supplementary Materials

The following are available online at https://www.mdpi.com/2075-4418/11/1/106/s1, Supplementary 1: Clinical data according to neurodevelopmental regression.
