# Supplementary material for: The Route to Autism Spectrum Diagnosis in Pediatric Practice in Bulgaria
Source: Diagnostics (Basel). 2021 Jan 11;11(1):106. doi: 10.3390/diagnostics11010106 (PMC7827839; doi:10.3390/diagnostics11010106)
Supplement: Supplementary file 1 [file diagnostics-11-00106-s001.pdf]

diagnostics

Scheme 1. Clinical data according to neurodevelopmental regression.

Variabe		Regression				No regression
		After infection	After vaccination	After other events	No preceding events	
		n = 8	*n* = 8	*n* = 6	*n* = 57	*n* = 107
Sex	male	8	5	3	45	87
	female	0	3	3	12	20
Abnormal pregnancy, birth or prematurity	yes	5	4	3	30	50
	no	3	4	3	26	56
	no data	0	0	0	1	1
Prematurity	yes	3	2	0	2	12
	no	4	6	6	47	89
	no data	1	0	0	8	6
Positive family hist.	yes	0	4	2	22	32
	no	8	4	4	30	70
	no data	0	0	0	5	5
Behavioral peculiarities—count	0	1	1	1	15	18
	1–3	2	2	2	16	40
	4–6	2	4	0	11	20
	no data	3	1	3	15	29
Age of first sings of neurodev. impair. [m.].	X	16,2	16,12	21	15,4	15,3
	min-max	12–29	12–24	12–36	8–30	7–36
Improvement after regression	yes	2	0	1	13	—
	no	6	8	3	34	—
	no data	0	0	2	10	-
Age of first sings of ASD [m.]	X	18,1	18,9	21,2	18,9	18,3
	min-max	12–24	12–40	17–36	15–60	9–84
Age of ASD diagnosis [m.]	X	44.4	38.8	54.7	36.7	40.4
	min-max	25-72	21-64	32-86	12-90	13-132
Delay in ASD diagnosis	X	28.1	20.5	32.7	20.16	24.25
	min-max	7–60	5–42	15–68	0-72	0–79
CARS	n	3	5	3	35	63
	X	41	39.5	37.5	36.9	37.3
	min-max	41.0–42.5	33.0–47.5	33.0–42.0	27.0–51.5	18.0–51.0
Etiology	idiop.	7	4	4	47	86
	syndr.	1	4	2	10	21
IQ/DQ	n	4	6	5	52	83
	X	58.9	52.4	45.7	53.6	54.7
	min-max	40.0–72.7	25.0–81.8	34.0–58.0	9.0–92.6	13.0–107.0
Language impairment	yes	8	8	6	57	104
	no	0	0	0	0	3
Epilepsy	yes	3	0	1	8	18
	no	5	8	5	49	89
Cerebral palsy	yes	0	1	0	1	2

	no	8	7	6	56	105

No data about presenece or absence of regression for 16 cases. No statistical difference between groups.
